# Fabrication and Characterization of Aligned Flexible Lead-Free Piezoelectric Nanofibers for Wearable Device Applications

**DOI:** 10.3390/nano8040206

**Published:** 2018-03-29

**Authors:** Sang Hyun Ji, Ji Sun Yun

**Affiliations:** Electronic Convergence Materials Division, Korea Institute of Ceramic Engineering and Technology, Jinju 52851, Korea; sanghyun_ji@kiect.re.kr

**Keywords:** lead-free piezoelectric nanofiber, aligned arrays, electrospinning, nanofiber composites, piezoelectric devices

## Abstract

Flexible lead-free piezoelectric nanofibers, based on BNT-ST (0.78Bi_0.5_Na_0.5_TiO_3_-0.22SrTiO_3_) ceramic and poly(vinylidene fluoride-trifluoroethylene) (PVDF-TrFE) copolymers, were fabricated by an electrospinning method and the effects of the degree of alignment in the nanofibers on the piezoelectric characteristics were investigated. The microstructure of the lead-free piezoelectric nanofibers was observed by field emission scanning electron microscope (FE-SEM) and the orientation was analyzed by fast Fourier transform (FFT) images. X-ray diffraction (XRD) analysis confirmed that the phase was not changed by the electrospinning process and maintained a perovskite phase. Polarization-electric field (P-E) loops and piezoresponse force microscopy (PFM) were used to investigate the piezoelectric properties of the piezoelectric nanofibers, according to the degree of alignment—the well aligned piezoelectric nanofibers had higher piezoelectric properties. Furthermore, the output voltage of the aligned lead-free piezoelectric nanofibers was measured according to the vibration frequency and the bending motion and the aligned piezoelectric nanofibers with a collector rotation speed of 1500 rpm performed the best.

## 1. Introduction

In the field of wearable electronic devices, there is a strong demand for portable smart devices that are not bulky and do not have to be too frequently charged. Thus, producers of wearable electronic devices are striving to create electronic devices that meet this demand. Piezoelectric modules are attractive as an emerging solution for this issue because they convert mechanical energy to electrical energy and harvest energy from motions of the human body [1,2]. For the application of wearable electronic devices, development of flexible and lead-free piezoelectric materials was necessary but lead zirconate titanate—Pb(Zr_x_Ti_(1−x)_)O_3_ (PZT)—piezoelectric ceramic materials that are most widely used for piezoelectric energy harvesting are brittle and contain harmful lead. On the other hand, flexible piezoelectric polymers such as polyvinylidenefluoride (PVDF) and P(VDF-trifluoroethylene(TrFE)), are poor in piezoelectricity and some other ways to improve the piezoelectric properties of PVDF nanofibers were studied [3]. In response to these challenges, composites of lead-free ceramics and polymers have been developed and development of nanofiber-based composites has also been studied to improve flexibility [4,5,6].

**Figure 1:**
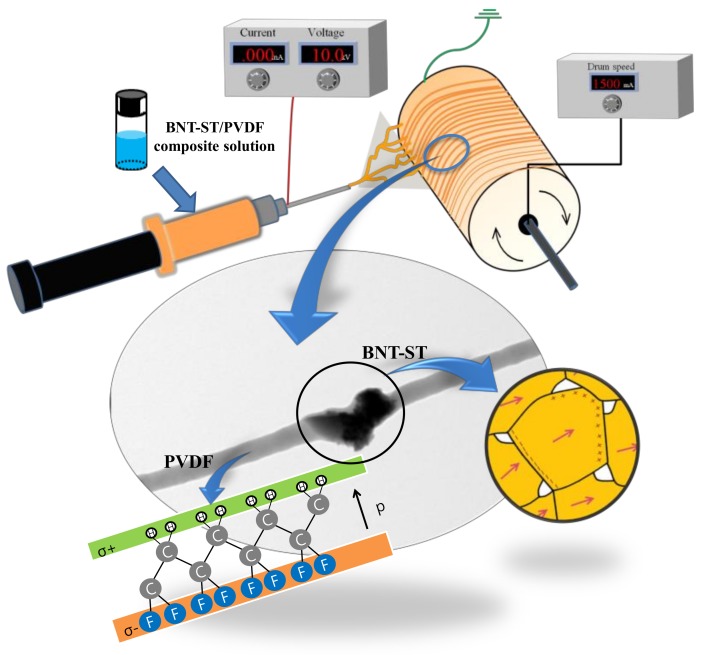
A schematic illustration of aligned dipoles in a polymer region and a ceramic region of the aligned piezoelectric nanofibers.

A study on improving piezoelectric properties of piezoelectric nanofibers was also done using the alignment process of electrospinning [7,8,9]. Particularly, when the piezoelectric nanofibers were aligned, both the output voltage and the output current increased over that of the randomly arranged nanofibers [9]. This was because the β-phase of the ferroelectric polymer in the nanofiber increased with the alignment of the piezoelectric nanofibers. The β-phase increase preferentially aligns the main molecular chains along the nanofiber longitudinal axis and at the same time improves the orientation of the piezoelectric active dipole (C-F) in a direction perpendicular to this axis. This is consistent with the preferred dipole arrangement along the direction of the resonant field during nanofiber collection and the piezoelectric properties seem to improve. However, the research on the alignment of piezoelectric nanofibers is limited to ferroelectric polymer nanofibers and the alignment of piezoelectric nanofibers of ceramic/polymer composites has not yet been reported. The piezoelectric properties of piezoelectric composite nanofibers seem to improve through the alignment process because not only the dipole of the polymer but also the dipole of the ceramic is aligned and poled along the electric field direction during the electrospinning process, as shown in Figure 1.

In this study, piezoelectric nanofibers of BNT-ST(0.78Bi_0.5_Na_0.5_TiO_3_-0.22SrTiO_3_)/PVDF-TrFE composites were fabricated by the electrospinning method and the effects of the alignment degree of the piezoelectric nanofiber were investigated according to the rotation speeds of the collector during electrospinning. The microstructure of the collected nanofibers was analyzed by field emission scanning electron microscope (FE-SEM) and the orientation was analyzed by fast Fourier transform (FFT) images. The crystal phase of the piezoelectric nanofibers was analyzed by X-ray diffraction (XRD) analysis and the piezoelectric properties were analyzed by Polarization-electric field (P-E) loops and piezoresponse force microscopy (PFM). The output voltage of the piezoelectric nanofiber modules according to the bending motion and vibration frequency was measured by an oscilloscope and the aligned piezoelectric performed the best.

## 2. Experimental Procedure

Lead-free BNT-ST piezoelectric ceramics powder were fabricated by a traditional solid-state reaction method. High purity Bi_2_O_3_, Na_2_CO_3_, SrCO_3_ and TiO_3_ were used as starting materials and all raw materials were purchased from Kojundo chemical (Saitama, Japan, purity > 99.9%). The BNT-ST ceramic particles were controlled to less than 100 μm by mash control. To prepare an electrospinning precursor solution, PVDF-TrFE copolymers with mole fractions of 75% VDF and 25% TrFE (Measurement Specialties, Hampton, USA), *N*,*N*’-dimethylformide (DMF, Sigma-Aldrich, St. Louis, MO, USA, 99.5%) and acetone (Sigma-Aldrich, St. Louis, MO, USA, 99.5%) were vigorously mixed at room temperature for 24 h. The weight ratio of each substance in the precursor solution was as follows: PVDF-TrFE:acetone:DFM = 2:5:5. After stirring, the BNT-ST ceramics powders were added to the precursor solutions in an amount of 60 wt % and the solutions were vigorously stirred for 24 h at room temperature to obtain a homogeneous solution.

The homogeneous precursor solution was loaded to a 10 mL plastic syringe with a metallic needle of 21 G and the needle was connected to a voltage power supplier. A positive high voltage of 10–15 kV was applied and the feeding rate of the precursor solutions was 1 mL/h. The nanofibers were electro-spun on the aluminum foil attached to a cylindrical drum, which was placed at a distance of 10 cm from the tip of the needle. To align the nanofibers, the rotation speed of the cylindrical drum was adjusted to 0 rpm, 500 rpm, 1000 rpm and 1500 rpm. All the experiments were conducted at room temperature under a relatively low humidity of 20–40%. 

After the electrospinning, the as-spun BNT-ST nanofibers with different rotation speeds were fabricated as piezoelectric modules with interdigitated copper electrodes between the bottom and top of the nanofibers by a Warm Isotropic press (WIP) process at 70 °C and 80 bar and then high voltages of 1 kV were applied to the fabricated piezoelectric modules at room temperature for 1 h for the poling process to improve of the piezoelectric performance [5]. 

A FE-SEM (JSM-6700F, Tokyo, Japan) was used for microstructure characterization of the BNT-ST nanofiber according to the rotation speed of the collector. The crystal structures of the BNT-ST nanofibers collected at the cylindrical drum with different rotational speeds were analyzed by XRD (max 2200V, Rigaku Corporation, Tokyo, Japan). The local piezoelectric response was characterized using PFM (NanostationII, SiS-GmbH, Kaiserstrasse, Germany). The output voltages as a function of frequency for the nanofibers with different collector rotation speeds were measured using a vibration exciter (Type 4809, Bruel & Kjaer, Nearum, Denmark), a function waveform generator (33220A, Agilent, California, USA), a bipolar amplifier (HAS 4014, Yokohama, Japan) and an oscilloscope (Wavejet322, LeCroy, New York, USA). The output voltages according to the bending motion of 1 Hz using a bending machine (SPG Co., Ltd., Incheon, Korea) were measured and recorded by an oscilloscope (Wavejet322, LeCroy, New York, NY, USA).

## 3. Results and Discussion 

**Figure 2:**
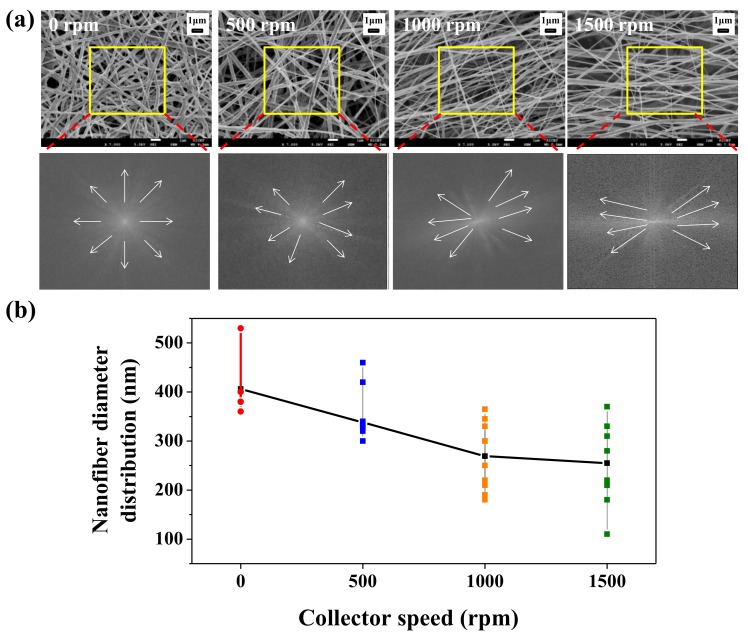
(**a**) Scanning electron microscope (SEM) images, Fast Fourier-transform (FFT) images and (**b**) nanofiber diameter distribution of BNT-ST nanofibers according to the rotation speed of the collector.

The SEM images and fast Fourier transform (FFT) images of the BNT-ST nanofibers according to the rotation speed of the collector are shown in Figure 2a,b shows the diameter distribution of the BNT-ST nanofibers calculated based on the SEM images. The FFT image shows grayscale pixels distributed in a pattern that reflects the degree of fiber alignment in the SEM image [8]. Although the BNT-ST nanofibers with no rotation of the collector showed a random array; as the rotation speed of the collector increased, the tendency to alignment of the BNT-ST nanofibers in one direction became stronger. In Figure 2a, the BNT-ST nanofibers with a 0 rpm rotation speed showed a random array in the SEM images and the FFT image generated from the original SEM images showed the grayscale pixels distributed in a symmetrical, circular shape. In contrast, the BNT-ST nanofibers with a rotation speed of 1500 rpm showed the aligned nanofibers in the SEM images and the FFT output image generated the grayscale pixels distributed in a non-random, elliptical distribution. In other words, the faster the rotation speed of the collector, the more the BNT-ST nanofibers were aligned in one direction and the FFT output image revealed greater elliptical distribution. In Figure 2b, the smaller average diameter and slightly broader diameter distribution of the BNT-ST nanofibers were observed with increasing the rotation speed of the collector. This is because the rotation speed of the collector induced tensile stresses on the nanofibers, before being spun around the collector [10,11]. 

**Figure 3:**
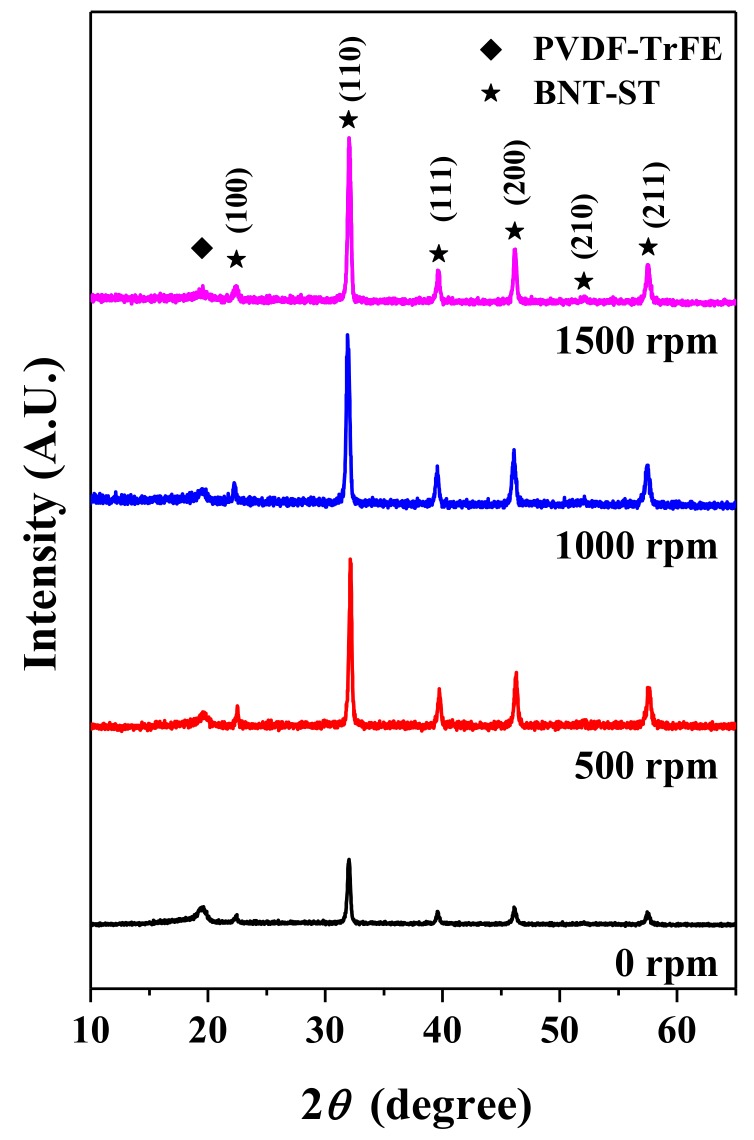
X-Ray Diffraction (XRD) patterns of the BNT-ST nanofibers according to the rotation speed of the collector.

Figure 3 shows the XRD patterns of the BNT-ST nanofibers according to the rotation speed of the collector. First, the XRD peaks at 2θ values of about 19.8°, which corresponds to the (110) reflections of the β phase of the PVDF-TrFE polymer, can be induced by an electrospinning process [12]. The non-polar α-phase of the PVDF-TrFE polymer is generally converted into the polar β-phase due to the electrospinning process and the piezo- and pyroelectric properties mainly result from the β-phase. As a result, the piezoelectric properties of polymer parts are improved by electrospinning. Second, five prominent peaks at 2θ values of about 32.7° (110), 40.3° (111), 46.8° (200), 52.7° (210) and 58.1° (211) corresponding to the perovskite crystal phase of BNT-ST [13] were observed in XRD patterns of the BNT-ST nanofibers with the different collector rotation speeds. This means that the crystal phase of the BNT-ST ceramics did not change with the electrospinning process and the β-phase peak of the piezoelectric polymer and the perovskite peaks of the piezoelectric ceramics coexisted in the nanofiber. Furthermore, as the rotation speed of the collector increased, the polar β-phase peak intensity of PVDF-TrFE was similar but the perovskite crystal peak intensity of BNT-ST was significantly increased. In other words, the β-phase of the piezoelectric polymer was not significantly affected by the rotation speed of the collector but the crystal phase of the BNT-ST ceramics was remarkably improved with increase in the rotation speed of the collector. From these results, it is expected that the piezoelectric performance will be improved as the rotation speed of the collector increases.

**Figure 4:**
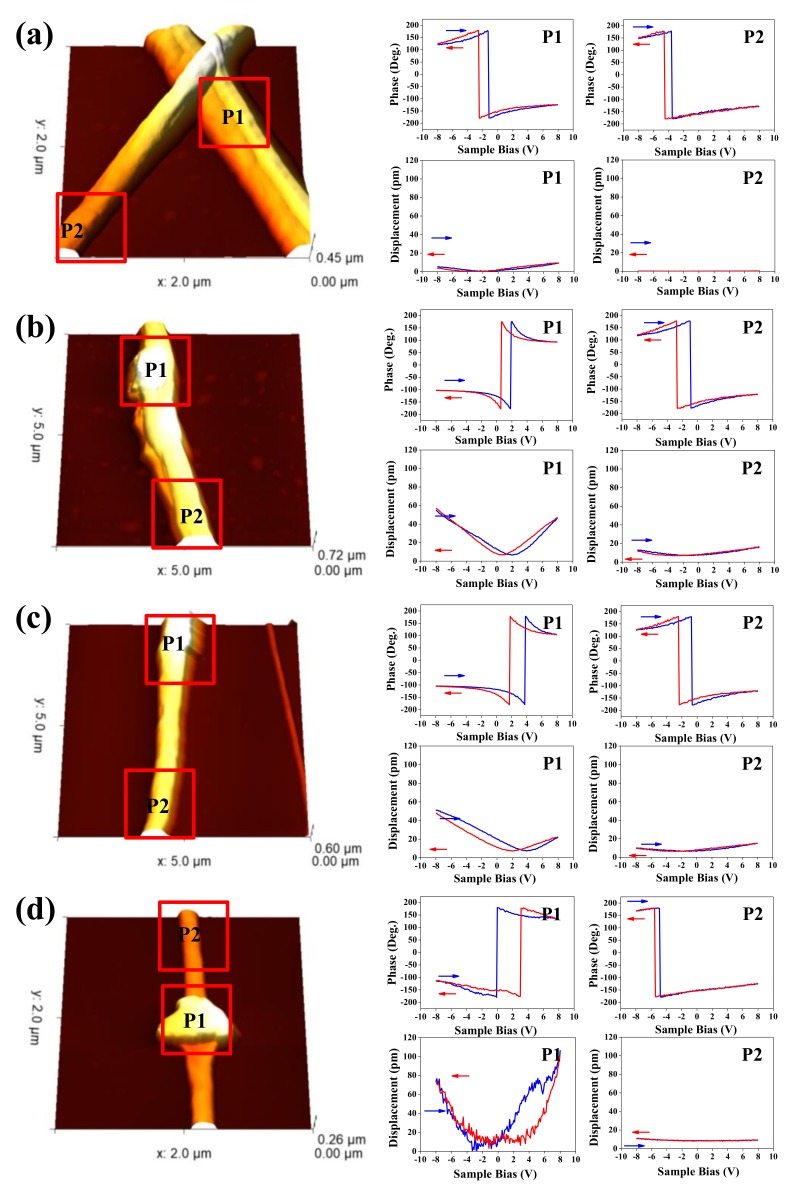
Piezoresponse force microscopy (PFM) images, phase hysteresis loops and amplitude hysteresis loops of the BNT-ST nanofibers according to the rotation speed of the collector: (**a**) 0 rpm, (**b**) 500 rpm, (**c**) 1000 rpm and (**d**) 1500 rpm.

To evaluate the nanoscale electromechanical response of the BNT-ST nanofibers with different collector rotation speeds, 3D topography images, phase hysteresis loops and amplitude hysteresis loops were analyzed by PFM as a function of applied DC voltage from −8 V to 8 V, as shown in Figure 4. In the PFM 3D topographic images, with more embedded BNT-ST ceramics, the images are brighter (or slightly protruding) and two areas of P1 (higher ceramic content area) and P2 (lower ceramic content area) were intensively analyzed. The PFM phase hysteresis loops of the BNT-ST nanofibers prepared at different rotation speeds of the collector are directly associated with the polarization switching of the P1 and P2 local regions and the phase changes of the phase hysteresis loops for all the BNT-ST nanofibers were about 180°, which indicated ferroelectric switching under an applied bias. The dipoles in both BNT-ST ceramic and P(VDF–TrFE) polymers seem to be poled along the electric field direction during the electrospinning process and both the P1 and P2 areas were likely to have aligned dipoles regardless of the rotation speed of the collector instead of randomly oriented dipoles [14]. In the amplitude (displacement) hysteresis loops, butterfly-shaped hysteresis loops, a typical strain-electric field (S-E) shape of a piezoelectric material [14,15], were observed at both the P1 area and P2 area of all BNT-ST nanofibers with different collector rotation speeds. The maximum strain (S_max_) and the longitudinal piezoelectric coefficient (d_33_) were calculated based on the amplitude hysteresis loops. For the BNT-ST nanofibers with a 0 rpm rotation speed, which has a random array in the SEM images in Figure 2, 16.95 pm of S_max_ and 13 pm/V of d_33_ were observed in the P1 area and 0.92 pm of S_max_ and 0.7 pm/V of d_33_ in the P2 area. On the other hand, the BNT-ST nanofibers at a rotation speed of 1500 rpm, which showed the aligned nanofibers in the SEM images in Figure 2, 192.87 pm of S_max_ and 148.1 pm/V of d_33_ were observed in the P1 area and 11.04 pm of S_max_ and 8.4 pm/V of d_33_ in the P2 area. These results mean that the BNT-ST ceramic phase had greater piezoelectricity than the P(VDF–TrFE) polymer phase and as the rotation speed of the collector increased, the BNT-ST nanofibers became aligned in the advancing direction and had a better piezoelectricity. Furthermore, the piezoelectric coefficient d_33_ (148.1 pm/V) of the aligned BNT-ST nanofibers at P2 was considerably larger than that of the reported values for the PVDF nanofibers (−58.5 pm/V) and PZT nanofibers (83.4 pm/V) [3,15] and the piezoelectricity of the nanofibers seemed to be improved by the alignment process during electrospinning.

**Figure 5:**
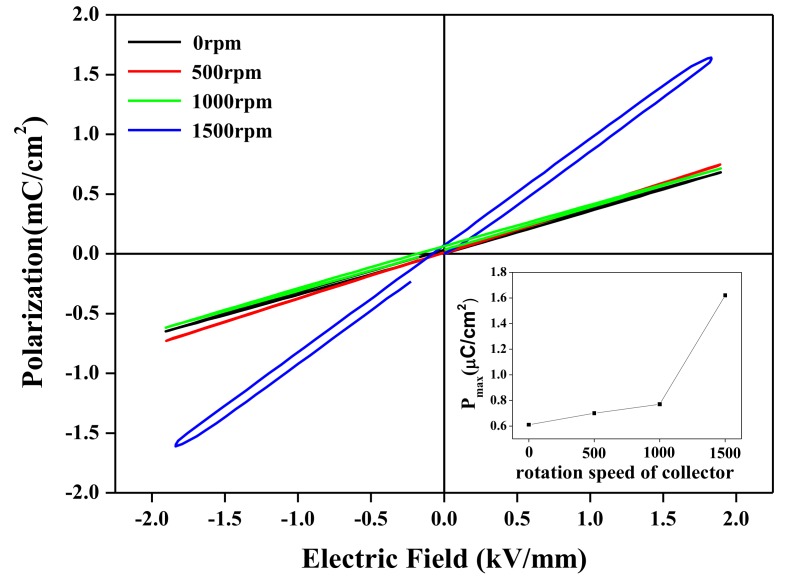
P-E hysteresis loops of the BNT-ST nanofiber modules according to the rotation speed of the collector. The inset shows maximum polarization values (P_max_) according to the rotation speed of the collector.

Figure 5 shows that the polarization versus electric field (P-E) hysteresis loops of the BNT-ST nanofiber modules with the bottom and top interdigitated copper electrodes were measured according to the rotation speed of the collector at room temperature, a frequency of 2 Hz and an applied voltage of 2 kV/mm. The inset graph of Figure 5 gives the P_max_ (maximum polarization) values calculated based on the P-E hysteresis loops of the BNT-ST nanofiber modules according to the rotation speed of the collector. As the rotation speed of the collector increased, the P_max_ values increased. Particularly, in the BNT-ST nanofiber modules with a rotation speed of 1500 rpm, which were the aligned nanofibers, the P_max_ value was 1.62 μC/cm^2^. This value was about 2.7 times higher than the P_max_ value (0.6 μC/cm^2^) of the randomly arrayed BNT-ST nanofiber modules with a 0 rpm rotation speed. On the other hand, it was confirmed that the P_max_ value of the BNT-ST nanofiber modules with the rotation speed of the collector from 0 rpm to 1000 rpm was slightly increased to from 0.6 μC/cm^2^ to 0.8 μC/cm^2^. These results confirmed that a slight increase in the alignment degree of the BNT-ST nanofibers does not contribute significantly to the piezoelectric performance and there is the sufficient alignment degree for the improved piezoelectric performance. In other words, as the rotation speed of the collector increased until over 1500 rpm, the aligned BNT-ST nanofiber modules showed a higher degree of polarization and better piezoelectric characteristics.

**Figure 6:**
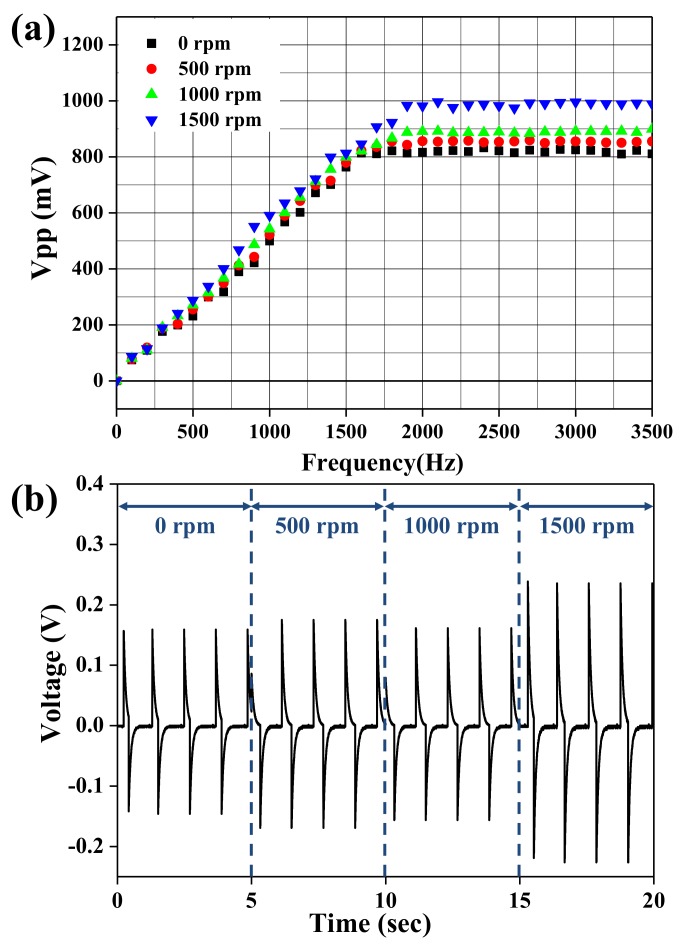
The output voltage according to (**a**) vibration frequency and (**b**) bending motion for the BNT-ST nanofiber modules with different collector rotation speeds.

Figure 6a shows the output voltages of the BNT-ST nanofiber modules with different collector rotation speeds according to vibration frequencies from 0 Hz to 3500 Hz. The output voltages of all the BNT-ST piezoelectric nanofiber modules improved with an increase in the vibration frequency and saturated at about 2000 Hz. The higher the rotation speed of the collector and the more aligned the BNT-ST nanofibers became and the output voltages according to the frequency for the BNT-ST nanofiber modules were higher. For the well-aligned BNT-ST nanofiber modules with a rotation speed of 1500 rpm, the saturated V_pp_ value at 2000 Hz was 0.99 V, which is about a 23.7% increase over the 0.80 V of the saturated V_pp_ of the randomly arranged BNT-ST nanofiber modules with a 0 rpm rotation speed. As the rotation speed of the collector increased, the saturated V_pp_ values at 2000 Hz increased. The output voltages of the BNT-ST nanofiber modules with different collector rotation speeds according to bending motion with 1 Hz are shown in Figure 6b. Although the randomly arranged BNT-ST nanofiber modules with a 0 rpm rotation speed had a V_pp_ value of 0.31 V, the well-aligned BNT-ST nanofiber modules with a rotation speed of 1500 rpm had a V_pp_ value of 0.56 V. As the rotation speed of the collector increased, the V_pp_ value according to the bending motion increased. These results indicate that when the piezoelectric nanofibers were well aligned in a certain direction by the increase in the rotation speed of the collector and the well-aligned BNT-ST nanofiber modules had better piezoelectric characteristics. The electric displacement in the BNT-ST nanofiber modules between adjacent electrode fingers of the interdigitated electrodes was parallel to aligned direction and as the polarized direction and the applied voltage direction match, we considered that the piezoelectric characteristics are maximized.

## 4. Conclusions

Flexible lead-free piezoelectric nanofibers based on BNT-ST ceramic and PVDF-TrFE copolymers were fabricated by an electrospinning method and the lead-free piezoelectric nanofibers were well aligned with a higher collector rotation speed. The microstructure, orientation and crystal structure of the lead-free piezoelectric nanofibers were observed by FE-SEM images, FFT images and XRD analysis. Compared to randomly arranged BNT-ST nanofiber modules, the well-aligned BNT-ST nanofiber modules demonstrated higher piezoelectric characteristics in the P-E hysteresis loop analysis and in the output voltage analysis according to vibration frequency and bending motion. In particular, the well-aligned BNT-ST nanofiber modules with a rotation speed of 1500 rpm had a saturated V_pp_ value of 0.99 V at 2000 Hz in the vibration frequency test and a V_pp_ value of 0.56 V in the bending motion. In other words, as the piezoelectric nanofibers became more aligned in a certain direction by the increase in the rotation speed of the collector, the better piezoelectric characteristics were achieved.
